# Effect of disease duration in a randomized Phase III trial of rintatolimod, an immune modulator for Myalgic Encephalomyelitis/Chronic Fatigue Syndrome

**DOI:** 10.1371/journal.pone.0240403

**Published:** 2020-10-29

**Authors:** David R. Strayer, Diane Young, William M. Mitchell

**Affiliations:** 1 AIM ImmunoTech Inc., Philadelphia, Pennsylvania, United States of America; 2 Vanderbilt University School of Medicine, Nashville, Tennessee, United States of America; TNO, NETHERLANDS

## Abstract

**Background:**

Rintatolimod is a selective TLR3 agonist, which has demonstrated clinical activity for ME/CFS in Phase II and Phase III double-blind, placebo-controlled, randomized, multi-site clinical trials.

**Methods and findings:**

A hypothesis-based *post-hoc* analysis of the Intent to Treat (ITT) population diagnosed with ME/CFS from 12 independent clinical sites of a Phase III trial was performed to evaluate the effect of rintatolimod therapy based on disease duration. The clinical activity of rintatolimod was evaluated by exercise treadmill tolerance (ETT) using a modified Bruce protocol. The ITT population (n = 208) was divided into two subsets of symptom duration. Patients with symptom duration of 2–8 years were identified as the Target Subset (n = 75); the remainder (<2 year plus >8 year) were identified as the Non-Target Subset (n = 133). Placebo-adjusted percentage improvements in exercise duration and the vertical rise for the Target Subset (n = 75) were more than twice that of the ITT population. The Non-Target Subset (n = 133) failed to show any clinically significant ETT response to rintatolimod when compared to placebo. Within the Target Subset, 51.2% of rintatolimod-treated patients improved their exercise duration by ≥25% (p = 0.003) despite reduced statistical power from division of the original ITT population into two subsets.

**Conclusion/significance:**

Analysis of ETT from a Phase III trial has identified within the ITT population, a subset of ME/CFS patients with ≥2 fold increased exercise response to rintatolimod. Substantial improvement in physical performance was seen for the majority (51.2%) of these severely debilitated patients who improved exercise duration by ≥25%. This magnitude of exercise improvement was associated with clinically significant enhancements in quality of life. The data indicate that ME/CFS patients have a relatively short disease duration window (<8 years) to expect a significant response to rintatolimod under the dosing conditions utilized in this Phase III clinical trial. These results may have direct relevance to the cognitive impairment and fatigue being experienced by patients clinically recovered from COVID-19 and free of detectable SARS-CoV-2.

**Trial registration:**

ClinicalTrials.gov: NCT00215800.

## Introduction

Myalgic Encephalomyelitis/Chronic Fatigue Syndrome (ME/CFS) is a debilitating disorder characterized by an incapacitating fatigue that is not improved by bed rest and is associated with a diverse combination of variable signs and symptoms [1–3]. A significant majority of patients are female [4–6]; this gender predisposition establishes ME/CFS as an important women’s health issue. The etiologic/pathogenic basis for ME/CFS is unknown, although the evidence indicates that it is multifactorial with a variety of microbial, hormonal, and immunological abnormalities linked to its pathogenesis and dependent upon genetic signatures [7]. Although controversial, the best available data suggests that patients with ME/CFS have a life span reduced by about 18 years due to an apparent early incidence of cancer, heart disease, and suicide [8, 9].

In the AMP-516 Phase III clinical trial, patients with severe ME/CFS demonstrated significant improvement in the primary endpoint, exercise treadmill tolerance (ETT), compared to placebo controls following the twice weekly for 40 weeks systemic administration [12] of the selective TLR3 dsRNA agonist, rintatolimod (Ampligen^®^) [10, 11]. A hypothesis based *post-hoc* analysis of ETT response in a subset of study patients was performed. This subset (n = 75), selected primarily on baseline ME/CFS symptom duration, has revealed ≥2 fold higher placebo-adjusted rintatolimod improvements compared to the ITT population (n = 208).

## Methods

The AMP-516 study was a prospective, double-blind, placebo-controlled, randomized, Phase III trial with equal parallel cohorts; it was conducted in accordance with the ethical principles of the Delaration of Helsinki at 12 sites in the United States to evaluate the safety and efficacy of rintatolimod in ME/CFS [12] with patient informed consent and IRB approval (IRB #1: Essex Institutional Review Board, Inc., 121 Main Street, Lebanon, New Jersey 08833, IRB #2: The UMDNJ–RWJMS IRB, 97 Paterson Street, New Brunswick, NJ 08903, IRB #3: Dean Institutional Review Board, 2711 Allen Boulevard, Middleton, WI 53562). This pivotal trial enrolled patients meeting both the original Holmes CDC 1988 diagnostic criteria [1] and the revised Fukuda 1994 CDC case definition [2]. Although post-exertional malaise (PEM) was not an absolute requirement of the CDC case definitions, the CDC case definitions had PEM as a symptom of the disease. In 2015, the Institute of Medicine (IOM) recommended that PEM be required for a diagnosis of ME/CFS and PEM is now widely accepted as a key symptom for ME/CFS. A Karnofsky Performance Score (KPS) of 40–60 indicating severe debilitation [12] was an inclusion criterion for the clinical trial. The design of the study, including endpoints, was reviewed by the FDA prior to its initiation. Many of the ME/CFS patients were unable to physically perform the standard Bruce exercise protocol (S1 Table, panel A) commonly used for the evaluation of cardiac function [13]. To insure patient safety, a modified Bruce ME/CFS protocol (S1 Table, panel B) was used, with reduced energy requirements similar to protocols (S1 Table, panel C) designed for the debilitated and elderly [14]. The primary endpoint was established as a change in ETT from baseline to Week 40 of the study [12]. In order to decrease the likelihood of spontaneous remissions, the AMP-516 protocol required a diagnosis of ME/CFS for ≥1 year, which was extended to ≥2 years for performing the *post-hoc* analysis.

To test the hypothesis that patients with a shorter duration of symptoms (illness) would respond better than patients with a longer duration of the disease, the total AMP-516 ITT population (n = 208) was divided into 2 subsets. Because the median duration of symptoms for the entire population was 8.5 years, we selected 8 years as the maximum duration since we wanted the subset with the shorter duration of illness to represent less than 50% of the total population. Accordingly, we stratified the subsets based on a 2–8 year duration of symptoms vs <2 and >8 years. In the *post-hoc* data analysis patients having onset of symptoms between 2 and 8 years, PEM lasting more than 24 hours and the ability to walk on a moving treadmill for longer than one minute but less than 16 minutes (Target Subset, n = 75) were compared with the remainder of the ITT Population (Non-Target Subset, n = 133). The data analyses employed SAS (Version 9.2) statistical software (Cary, NC). Analysis of the raw ETT data showed consistency with normality and equality of the variances between treatment groups. Therefore, no transformation was performed (skewness was -0.16 for all patients), -0.18 for rintatolimod, and -0.19 for placebo). All statistical analyses were two-sided. Exercise treadmill duration and vertical rise were analyzed using the two-sided Student’s T-test. Both the Pooled Test for equal variances and the Satterthwaite Test for unequal variances were calculated. If the Equality of Variance Test indicated that there was a significant difference between the two variances, the result from the Satterthwaite Test was reported. If there was no significant difference, the result from the Pooled Test was reported. Comparison of the proportion of patients who improved ETT by at least 25% was analyzed by the Chi-squared test. Multivariable regression models were used to analyze the possible confounding factors of age, sudden onset, PEM, and gender on ETT response.

Study participants were required to undergo ETT testing using the modified Bruce protocol, which incorporated progressive increases in the treadmill inclination/grade from 0% to 21% in seven separate increments of 3% (S1 Table, panel B). The vertical rise component of the ETT testing protocol was calculated for each of the inclination stages completed. The last stage attempted, which was only partially completed in most cases, was also included in the calculation based on the percentage of completion. The increase in vertical rise from baseline to Week 40 was calculated for each patient and was expressed as vertical feet ascended (vertical rise).

## Results

The Target Subset of patients (n = 75) primarily identified as having a 2–8 year duration of symptoms had twice the placebo-adjusted percent increase in ETT response of the entire ITT population (n = 208). The remainder, identified as the Non-Target Subset (n = 133), failed to show any clinically significant ETT response to rintatolimod when compared to placebo.

The baseline demographics of the Target and Non-Target AMP-516 subsets are shown in Table 1. Mean age, gender, sudden onset and KPS were well matched between the subsets and the original ITT population. However as expected, a significant difference was observed between the two subsets with regard to the duration of ME/CFS baseline symptoms (p<0.001). The Target Subset had a mean duration of ME/CFS symptoms of 5.0±1.6 years for the rintatolimod cohort and of 4.9±1.9 years for the placebo cohort; in contradistinction, the Non-Target Subset had mean durations of 12.5±4.8 and 12.0±6.2 years, respectively, for the rintatolimod and the placebo cohorts. The ME/CFS patients enrolled into this trial had severe debilitation, with a median KPS of 50 (S2 Table), indicating a requirement for considerable daily assistance to care for their daily activities (S2 Table).

**Table 1:** Comparison of baseline demographic and disease characteristics of AMP-516 ITT population to the target and non-target subsets.

Parameter	ITT Population (n = 208)		Target Subset (n = 75)		Non-Target Subset* (n = 133)	
	Rintatolimod	Placebo	Rintatolimod	Placebo	Rintatolimod	Placebo
Number of ME/CFS patients	100	108	41	34	59	74
Duration of ME/CFS symptoms mean±SD (years)	9.5±5.3	9.7±6.2	5.0±1.6	4.9±1.9	12.5±4.8	12.0±6.2
Age†	43±9.3	43±10.1	41±9.4	41±9.9	45±9.0	45±9.9
% Female	70%	77%	71%	79%	70%	76%
% Sudden onset	62%	64%	61%	56%	63%	67%
Baseline KPS (mean±SD)	49.4±5.3	49.7±5.0	49.4±4.8	50.0±5.1	49.5±5.7	49.5±5.0
Baseline KPS (median±IQR)	50±6.7	50±6.7	50±3.3	50±2.5	50±6.7	50±6.7

*Non-Target Subset consists of the remainder of AMP-516 ITT Population that is not included in the Target Subset.

^†^Mean age in years.

Table 2 illustrates the significant difference between the Target and Non-Target Subsets in treadmill endurance. The difference between the rintatolimod and placebo cohorts in the Target Subset was 122.5 seconds, compared to 30.1 seconds in the Non-Target Subset and 67.5 seconds in the total ITT population. The difference in ETT duration was statistically significant for the ITT population and the Target Subset.

**Table 2:** Comparison of change from baseline in mean ETT duration at Week 40 for ITT population vs. target and non-target subsets.

AMP-516 Cohort	Increase from baseline (in seconds) mean±SD (95% mean CI)		% Increase in intra-group means	
	Rintatolimod	Placebo	Rintatolimod	Placebo
**ITT Population** n = 208	95.7±251.3 (45.8, 145.5)	28.2±226.5 (-15.0, 71.5)	16.6	4.8
	Δ = 67.5 p = 0.043*		Δ = 11.8	
**Target Subset** n = 75	146.7±261.1 (64.3, 229.1)	24.2±262.8	27.8	4.2
	Δ = 122.5 p = 0.047*		Δ = 23.6	
**Non-Target Subset** n = 133	60.2±240.2 (-2.4, 122.8)	30.1±209.7 (-18.5, 78.7)	9.8	5.1
	Δ = 30.1 p = 0.44*		Δ = 4.7	

Δ = Difference between rintatolimod and placebo.

* Student’s T-test (2-sided).

Table 2 also reveals the percent increase in intra-group mean exercise duration from baseline to Week 40. The placebo-adjusted mean increase seen in the Target Subset (Δ = 23.6%) is twice that seen for the ITT population as a whole (Δ = 11.8%). The placebo-adjusted mean increase seen in the Non-Target Subset (Δ = 4.7%) was not statistically significant. Despite fewer patients and reduced statistical power compared to the ITT population (n = 208), the placebo-adjusted mean increase in ETT (Δ = 23.6) within the Target Subset (n = 75) was statistically significant.

**Fig 1:**
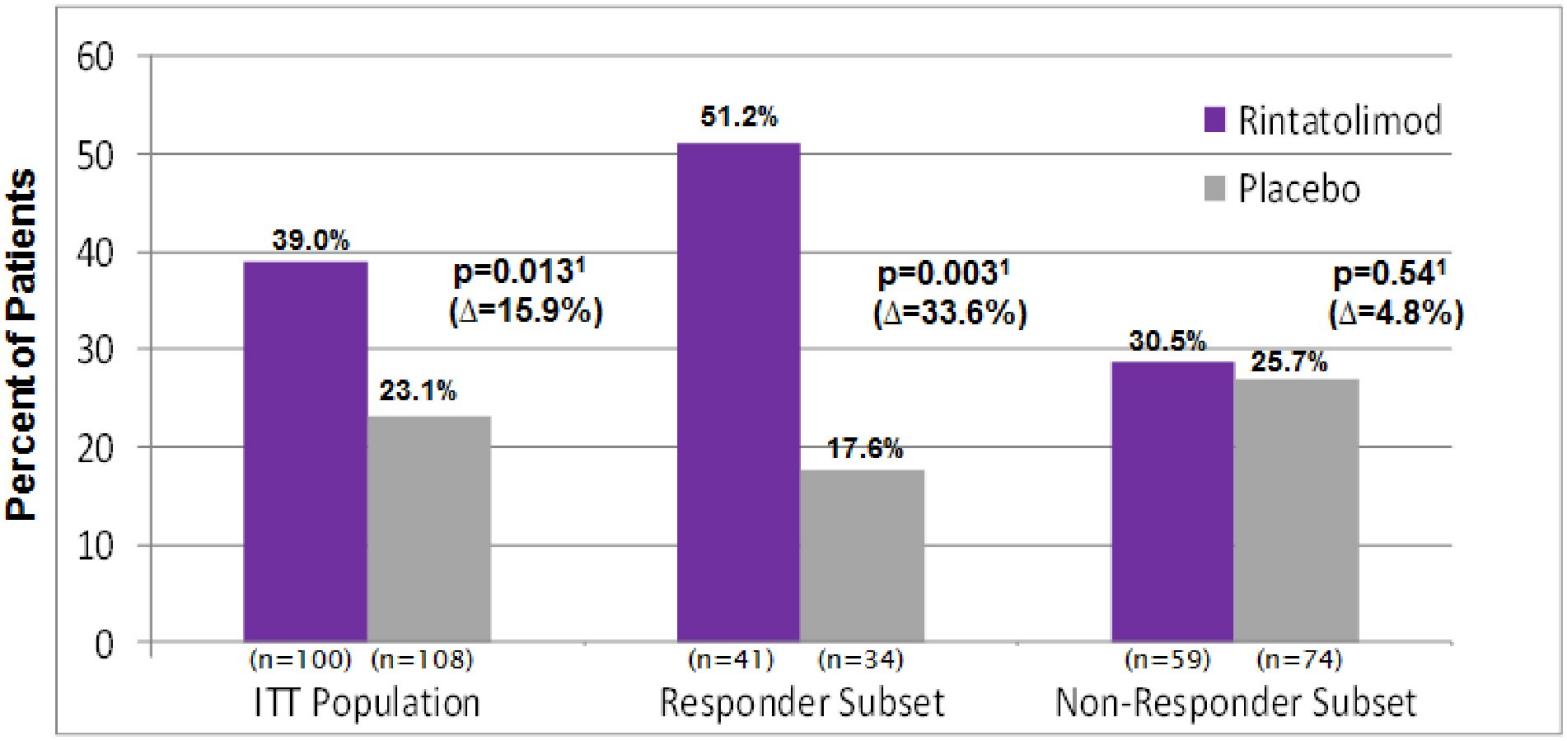
ME/CFS patients with a ≥25% increase in treadmill ET from baseline at Week 40.

A value of a ≥25% increase in ETT improvement was used as a high-bar element of our Target subset analysis. This was based on a request from the FDA (Division of Antiviral Drug Products) to establish a percent change in the clinical protocol that was above the variability of the intra-patient exercise tolerance at baseline. The percentage of ETT responders (*i*.*e*., exhibiting ≥25% improvement in exercise duration) seen in the rintatolimod cohort (39.0%) vs. placebo (23.1%) was statistically significant for the ITT population (p = 0.013). The majority of the patients receiving rintatolimod in the Target Subset (51.2%) were ETT responders vs placebo (17.6%) (p = 0.003). In Fig 1, the placebo-adjusted difference in percent responders (shown as the Δ value below the p-value in the figure) for the Target Subset is twice that seen for the overall ITT population (Δ = 33.6% vs Δ = 15.9%). There was only a 4.8% difference seen in Fig 1 between the rintatolimod and placebo cohorts for the Non-Target Subset, which was not significant (p = 0.54).

S1 and S2 Figs show each individual patient’s disease duration at baseline, along with their corresponding percent improvement in ETT from baseline for the Target and Non-Target Subsets, respectively, visually showing results in Fig 1.

Multivariable regression models were used to analyze the possible confounding factors of age, sudden onset, PEM, and gender on ETT response. Age, sudden onset, and PEM were found not to be confounding factors, while gender was a confounding factor with male patients responding better than females. Fortunately, both of the two subsets, the Target (2–8 year) and Non-Target (<2 and >8 year) subsets, as well as the entire ITT Population, were each well-balanced with no significant gender differences found when comparing their rintatolimod vs. placebo cohorts. Within the 2–8 year subset, the response rates for improving ETT duration by ≥25% for patients receiving rintatolimod were 44.8% (13/29) for females (p = 0.035) and 67.7% (8/12) for males (p = 0.057) compared to placebo. The percent of patients with ≥25% ETT improvement was 2.4 fold (females) and 4.7 fold (males) greater for the rintatolimod cohort compared to placebo. There was no significant difference in ETT response between the rintatolimod and placebo arms for either the female or male cohorts in the non-Target (<2 and >8 year) subset.

The ETT protocol included progressive increases in treadmill grade from 0% to 21% in seven increments of 3% (S1 Table, panel B). The vertical rise in feet was calculated for each patient. Table 3 documents the mean change in vertical rise from baseline to Week 40 for the ITT population and for each subset. The improvement in vertical rise in the ITT population was significantly greater (p = 0.033) for the rintatolimod cohort (56.9 feet) when compared to the placebo cohort (22.5 feet).

**Table 3:** Comparison of change from baseline in mean ETT vertical rise* (feet) at Week 40 for ITT population vs. the target and non-target subsets.

AMP-516 Cohort	Increase from baseline (vertical feet) mean±SD (95% mean CI)		% Increase in intra-group means	
	Rintatolimod	Placebo	Rintatolimod	Placebo
**ITT Population** n = 208	56.9**±**132.4 (30.7, 83.2)	22.5**±**93.0 (4.8, 40.3)	47.1	18.5
	Δ = 34.4 p = 0.033*		Δ = 28.6	
**Target Subset** n = 75	85.2**±**151.0 (37.6, 132.9)	23.3**±**110.5 (-15.3, 61.8)	91.0	20.7
	Δ = 61.9 p = 0.050*		Δ = 70.3	
**Non-Target Subset** n = 133	37.3**±**115.0 (7.3, 67.3)	22.2**±**84.6 (2.6, 41.8)	26.6	17.6
	Δ = 15.1 p = 0.401†		Δ = 9.0	

Δ = Difference between rintatolimod and placebo.

* for the definition of vertical rise, see last paragraph in Methods.

^†^ Student’s T-test (2-sided).

The increase in vertical rise seen for the Target Subset was 85.2 feet for the rintatolimod cohort vs. 23.3 feet for the placebo cohort. The difference in vertical rise was 61.9 feet and was at the threshold for statistical significance (p = 0.050). Nonetheless, the difference in percent increase in intra-group means between the rintatolimod and placebo cohorts seen for the Target Subset of 70.3% was over twice that seen for the ITT population of 28.6%. The increases in rise for the rintatolimod and placebo cohorts in the Non-Target Subset were 37.3 and 22.2 feet, respectively, however, this difference of 15.1 feet between the rintatolimod and placebo cohorts was not statistically significant (p = 0.401).

## Discussion

This *post-hoc* analysis of the successful AMP-516 double-blind, placebo-controlled, randomized, Phase III trial has identified a subgroup of patients defined primarily by the length of ME/CFS symptoms (2–8 years) with an increased likelihood of a clinically beneficial response to rintatolimod. Patients enrolled in the Phase III clinical trial AMP-516 met both the original CDC 1988 [1] diagnostic criteria and the revised 1994 CDC case definition [2]. An international consortium proposed in 2011 that Myalgic Encephalomyelitis was a preferable term for the syndrome complex [3]. In 2015, the Institute of Medicine (IOM) recommended that PEM be required for a diagnosis of the disease [15]; we have incorporated PEM as a requirement for the Target Subset, although only one patient was excluded from the Target Subset because of a lack of PEM. The percentage of patients with PEM was well-balanced with no significant differences seen between the rintatolimod and placebo cohorts within the ITT population and within both of the subsets (Target and Non-Target). The CDC 1988 case definition [1] defines “Description of the main symptom complex as initially developing over a few hours to a few days” as Minor Symptom Criteria number 11, which is commonly known as “sudden onset”. In AMP-516, 63.0% of the ITT population reported sudden onset of their illness. The proportion of rintatolimod patients improving ETT by ≥25% was statistically similar for those with sudden onset (38.7%) and for those with slow onset (39.5%).

### Debilitating fatigue

**Fig 2:**
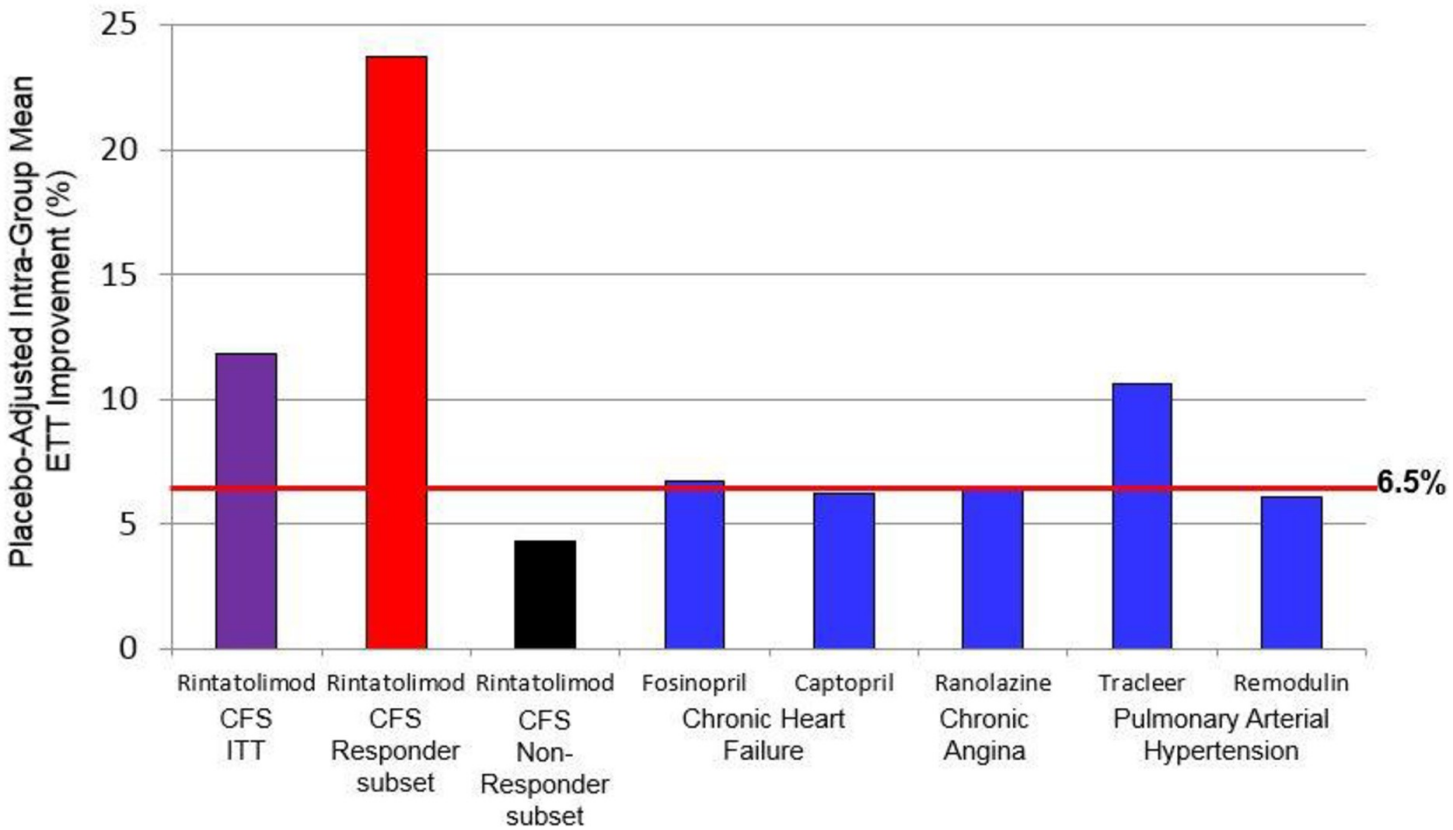
The Placebo-adjusted percent intra-group mean exercise improvements for rintatolimod: ITT population and the target subset comparisons to drugs approved for non-ME/CFS severe exertional fatigue.

The symptom common to all cases of ME/CFS is fatigue. For severe ME/CFS, the fatigue is debilitating. Cardiopulmonary exercise tolerance testing (ETT) is an objective measure of efficacy for physical fatigue and is accepted as a regulatory standard for approval of drugs ameliorating exertional fatigue. An improvement of ≥6.5% was based on prior increases in exercise tolerance recognized by the FDA for drugs ameliorating fatigue in non-ME/CFS indications. The AMP-516 Phase III protocol pre-specified a ≥6.5% improvement in intra-group mean exercise tolerance as demonstrating efficacy of rintatolimod in ME/CFS. A total of five drugs have been approved by the FDA based, in part, on improvement in exercise tolerance for indications that include congestive heart failure [16, 17], chronic angina [18, 19], and pulmonary hypertension (6 minute walk) [20, 21]. Four of the five approved agents provided approximately 6.5% improvement in placebo-adjusted exercise tolerance (Fig 2). Rintatolimod elicited an 11.8% improvement for the ITT study population for this exercise parameter and was similar in improvement to Tracleer (approved for pulmonary hypertension). The Target Subset within the ITT population (with symptoms between 2–8 years) had an increased response to rintatolimod with 23.6% improvement, which is over twice the clinical improvement/quality of life benefit observed for Tracleer and over three-fold that of any of the other 4 drugs approved by the FDA.

**Fig 3:**
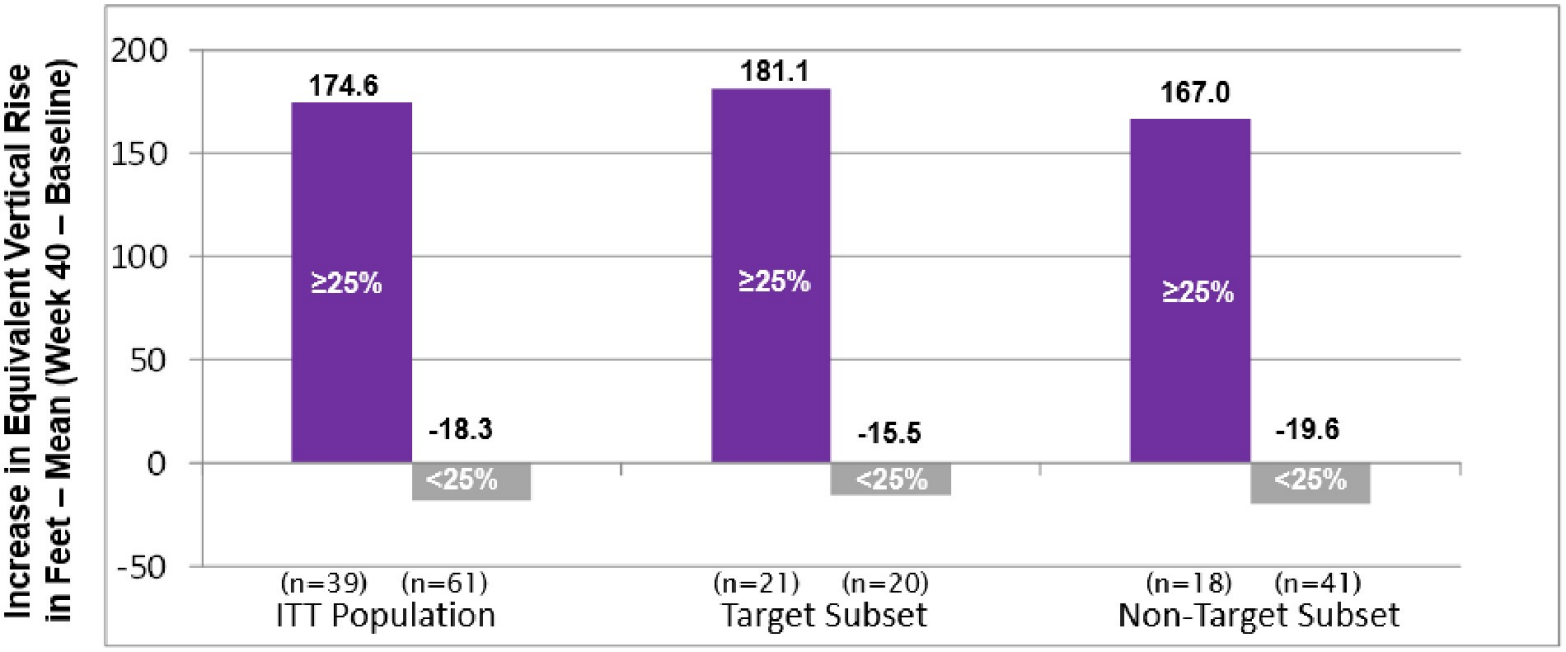
Rintatolimod-treated patients by ETT response (≥25% vs. <25%): Comparison of change from baseline in vertical rise at Week 40 for the ITT population vs. each subset.

A majority (51.2%) of patients in the 2–8 year Target Subset improved their ETT by ≥25%. But, in a real-life sense, just what does a ≥25% improvement in ETT duration mean? For example, what magnitude of improvement occurred for severely debilitated ME/CFS patients to increase ≥25% in ETT duration? Fig 3 shows the ETT response (≥25% vs. <25%) of rintatolimod-treated patients, comparing change from baseline to Week 40 in vertical rise for the ITT population and for each subset. For the entire rintatolimod treated ITT population, 39% of the patients improved ETT by ≥25%; those patients were able to ascend the equivalent of 174.6 more vertical feet at Week 40 as compared to baseline. This increase in ~175 vertical feet was, on average, accomplished over approximately 6–8 additional minutes on the treadmill at inclinations between 12–21%. The patients who did not improve ETT by at least 25% had a mean decrease in vertical rise of 18.3 feet compared to baseline. Similar results were seen for the Target Subset, with 51.2% of these patients improving ETT by ≥25% (181.1 vertical feet). Even patients in the Non-Target Subset with ≥25% ETT improvement in the rintatolimod arm were able to ascend about 167 additional vertical feet, similar to the ITT population. Rintatolimod treatment significantly increased the number of these responders in the ITT population (p = 0.013) and in the Target Subset (p = 0.003) when compared to placebo (Fig 1), demonstrating a substantial reduction in the profound and universal fatigue of severe ME/CFS and a corresponding significant improvement in quality of life.

### Secondary quality of life indicators

Although fatigue is a universal symptom which can be quantified by ETT, a number of symptoms and signs including cognitive impairment, sleep disturbance, dysautonomia, neuroendocrine abnormalities and PEM [22, 23] are observed with variable penetrance in ME/CFS. The rintatolimod subset of the ITT population exhibiting a ≥25% improvement in exercise duration also demonstrated a correspondingly significant improvement in two quality of life secondary clinical endpoints, KPS and Vitality Score within the Short Form-36 (SF-36), when compared to the <25% cohort [24]. The patient self-evaluated Vitality Score increased from 9.49 at baseline to 24.10 at week 40, a 14.6 point increase (p = 0.008), and almost three times the minimum clinically important difference (MCID) of 5 points [24]. The Vitality Score is considered to be one of the best SF-36 subscales for measuring the reduction in function seen in patients with ME/CFS [25]. A similar statistically significant improvement was seen with the physician evaluated KPS. The median KPS increased from 50 to 60 (p = 0.005). A 10-point increase in KPS was pre-specified as a clinically significant improvement in the AMP-516 protocol reviewed by the FDA prior to authorizing the study to proceed. A KPS of 50 requires considerable assistance from a caregiver to complete common daily activities (*i*.*e*., bathing, dressing, grooming, food preparation, eating, *etc*.); at a KPS of 60, a patient requires occasional assistance (once or twice weekly) for these same daily activities (S2 Table). Importantly, the 10 point increase in KPS and the 14.6 point increase in Vitality Score are both clinically relevant, representing objective and decisive improvements in quality of life [24, 26].

### Safety

Over 90,000 doses of rintatolimod have been administered by intravenous infusion across all clinical trials and have been generally well-tolerated. Safety data from the two pivotal clinical trials (Phase 2 and Phase 3) in severely debilitated ME/CFS patients with KPS ≤60 (AMP-502 and AMP-516) show that the number of serious adverse events (SAEs) associated with rintatolimod is no different than placebo-related SAEs [7]. The most frequent adverse event is a limited flu-like syndrome (consisting of headache, chills, fever, flushing, and myalgia) that occurs in approximately 44–45% of rintatolimod patients vs. 30–33% of placebo patients.

### Rintatolimod mechanism of action

The Toll-Like Receptors (TLRs) act as a first line of defense against microbial pathogens by the induction of innate immunity; they further provide the initial cellular orchestration for the induction of adaptive immune responses to provide specific humoral and cellular immunity mediated in part by inflammatory cytokines [27]. TLRs are abundant in dendritic cells, central to the host adaptive immune response system [28, 29]. All of the TLRs employ an inflammatory MyD88-dependent signaling pathway with the exception of TLR3 that utilizes the MyD88-independent TRIF pathway [30]. Two other dsRNA inducers of gene expression that initiate innate immune responses are the cytosolic helicases, MDA5 and RIG-1, which activate the inflammatory cytokine-inducing mitochondrial antiviral signaling protein (MAVS) [31].

The introduction of a non-paired uridine into the polycytidine chain of rintatolimod as Poly I:Poly C_12_U creates a mismatched region of thermodynamic instability in the dsRNA, restricting the activity of rintatolimod to that of a TLR3 agonist [10, 11]. The importance of this unique property of rintatolimod as a selective TLR3 agonist is a reduction of the systemic inflammatory cytokines [32] that have limited the clinical utility of other TLR3-activating ligands, such as Poly I:Poly C and viral dsRNA, that also activate MDA5 and RIG-1 [33]. Rintatolimod (Poly I:Poly C_12_U) induction of innate and adaptive immunity is restricted to TLR3 [10, 11, 33]. This restriction of rintatolimod to TLR3 is responsible for the absence of systemic cytokine induction in primates, including humans [32]. Of significance to the aberrant immune responses observed in ME/CFS [34, 35] is a recent seminal observation in cancer that rintatolimod increases the ratio of Teff/Treg cells in the tumor microenvironment, in contrast to non-restricted dsRNA TLR3 agonists [33]. This provides a relative increase in the killer T-cell balance between immune rejection and tolerance. Other TLR-activating agonists that employ the systemic inflammatory cytokine-inducing, MyD88-dependent or MAVS signaling pathways, inextricably engender greater levels of toxicity when compared to rintatolimod. The potential role of chronic stress mediated by post-infection damaged-associated molecular patterns (DAMPs) in genotypes at-risk and its association with chronic hyper-activation of NFκB has been recently reviewed by Morris et al. [36]. The selective activation of TLR3 by rintatolimod through the MyD88-independent/NFκB minimizing TRIF pathway is believed to be responsible for its generally well-tolerated clinical safety profile. The presence of DAMPs in ME/CFS has been reported by multiple investigators and epigenetic mechanisms in immune response genes play an important role in the development of DAMPs in individual patients post-infection [36].

### Time dependent efficacy of rintatolimod in severe ME/CFS

Early in the course of ME/CFS there are definite patterns of plasma cytokine activation that are not seen in subjects with longer disease durations [34]. Indeed, a higher correlation of these cytokine signatures was found for illness duration than for illness severity. With short illness duration (≤3 years) levels of IL-1a, IL-8, IL-12p40, IL-17A, and TNFα were significantly higher (p<0.03) when compared to subjects with a longer duration of illness. Russell et al. (2016) [37] also studied cytokine expression as a biomarker in ME/CFS as a function of disease duration. Levels of IL-1a were high in adolescent subjects with recent onset of ME/CFS and progressively decreased with increased illness duration. Similarly, high levels of IL-8 in early ME/CFS dropped in subjects with the illness for more than 2 years. In contrast, in subjects 18 years and older, low levels of IL-6 were found in early ME/CFS, while the opposite was seen after more than 2 years of the disease. These results suggest that the immunopathology of ME/CFS is not static, but changes over time. The mechanism for the ETT improvement seen in the rintatolimod cohort compared to placebo as a function of baseline disease duration is not known. Time-dependent epigenetic-based gene regulation including cytokine expression provides an attractive potential mechanism.

### Epigenetic components in ME/CFS

Genomic polymorphisms [38] affecting expression or function of ME/CFS associated genes [39–42] in a time-dependent manner may be influenced by time-dependent epigenetic mechanisms of DNA methylation, microRNAs, and transposable elements.

#### DNA methylation

Cytosine methylation at CpG dinucleotide genomic sites regulates gene expression without disrupting the nucleotide sequence. Differential DNA methylation in promoter regions has been reported in greater numbers of ME/CFS patients versus matched controls. These methylation patterns occurring on gene coding regions as well as promoters and regulatory elements suggest a dysregulation of the immune system in ME/CFS [43].

#### MicroRNAs(miRNA)

Non-coding DNA provides the origin of miRNAs which serve as ~22-nucleotide “sculptors” of gene regulation. Although miRNAs principally inactivate target mRNA, most commonly by binding to the RNA 3’UTR with resultant target mRNA degradation, 5’UTR or promoters are infrequent targets. Extracellular miRNAs act in cell-to-cell communication as chemical messengers [44]. The differential expression reported in several studies in ME/CFS patients is possibly limited by the use of differing ME/CFS case definitions [45]. The reported differential levels of miRNA based on gender in ME/CFS [46] may explain our observed gender differences in the magnitude of ETT responses to rintatolimod.

#### Transposable elements (TE)

Non-coding DNA provides the source of repetitive TE, which independently replicates and translocates to new locations within the genome. The vast majority are retrotransposons, which replicate by reverse-transcription into DNA and are inserted into other loci within the cell genome. The most common repetitive elements are two families denoting long interspersed repetitive element (LINE) and short interspersed repetitive element (SINE). Next Generation Sequencing (NGS) frequently masks SINE sequences in wide genome sequencing requiring directed reporting of these repetitive elements. LTR elements are a third type of retrotransposition resembling the integrated form of proviruses. Although abundant in number (8% of the human genome), most are defective, restricted to vertical transmission, and are non-infectious, although there are some exceptions. Human endogenous retroviruses (HERV) transcription has been reported for organ-specific (multiple sclerosis) and systemic (lupus erythematosus) autoimmune mediated diseases. HERV expression in human PBMC has been associated with immune responses [47]. External factors, such as infections and stress, have been shown to have an ability to activate TE-associated epigenetic control mechanisms. ME/CFS is associated with several stressors, including altered cytokine profiles and infections [45]. It is possible that aberrant TE activation could also be playing a pathogenic role in ME/CFS.

### Drugs in development

Rintatolimod is the only drug to have completed successful advanced placebo-controlled clinical trials (Phase II/III) for ME/CFS and is approved for severe ME/CFS in Argentina. To the best of our knowledge, there are no other drugs with a NDA for ME/CFS under review at the FDA or other international regulatory bodies. Rituximab, a monoclonal antibody which binds to CD20 expressed on B-cells, was originally reported in open-label trials [48, 49] and in a small double-blind placebo trial as being active in ME/CFS [50]. However, a 152-patient, double-blind, placebo-controlled clinical trial in Norway concluded that B-cell depletion using several infusions of rituximab over 12 months was not associated with clinical improvement in patients with ME/CFS [51, 52]. Significantly, and in contrast to rintatolimod, rituximab was associated with increased drug related serious adverse events compared to placebo.

### Relationship to COVID-19

There is increasing creditable anecdotal evidence that patients recovering from COVID-19 can develop a ME/CFS-like illness [53, 54]. Individuals with this condition have been referred to as COVID-19 “long-haulers”. This post-viral syndrome can be incapacitating with brain fog, fatigue, and difficulty concentrating and is reported to last for many weeks following COVID-19 clinical recovery and clearance of SARS-CoV-2. Whether rintatolimod would have any beneficial activity if administered early to patients with this condition needs to be investigated.

## Conclusion

The subset of patients reported herein represents a population with onset of symptoms between 2–8 years prior to the initiation of therapy for severe ME/CFS. The majority of these patients (51.2%) receiving rintatolimod substantially improved physical performance and quality of life. Moreover, there is a time-sensitive window for expected rintatolimod efficacy under the conditions employed in the clinical trial. Whether Non-Targets could benefit from a treatment duration longer than 40 weeks, or from rintatolimod combined with other drugs, or whether clinically recovered, SARS-CoV-2 negative COVID-19 debilitated patients with “long hauler” post-viral syndrome would benefit from rintatolimod are topics for further investigation.

## Supporting information

S1 Fig Percent change in ETT at Week 40 for the target subset based on baseline CFS duration. (DOCX)

S2 Fig Percent change in ETT at Week 40 for the non-target subset based on baseline CFS duration. (DOCX)

S1 Table Comparative treadmill exercise testing protocols. (DOCX)

S2 Table Clinical significance of Karnofsky Performance Scale* scores. (DOCX)
