# Supplementary figures and images for: Effect of disease duration in a randomized Phase III trial of rintatolimod, an immune modulator for Myalgic Encephalomyelitis/Chronic Fatigue Syndrome
Source: PLoS One. 2020 Oct 29;15(10):e0240403. doi: 10.1371/journal.pone.0240403 (PMC7595369; doi:10.1371/journal.pone.0240403)

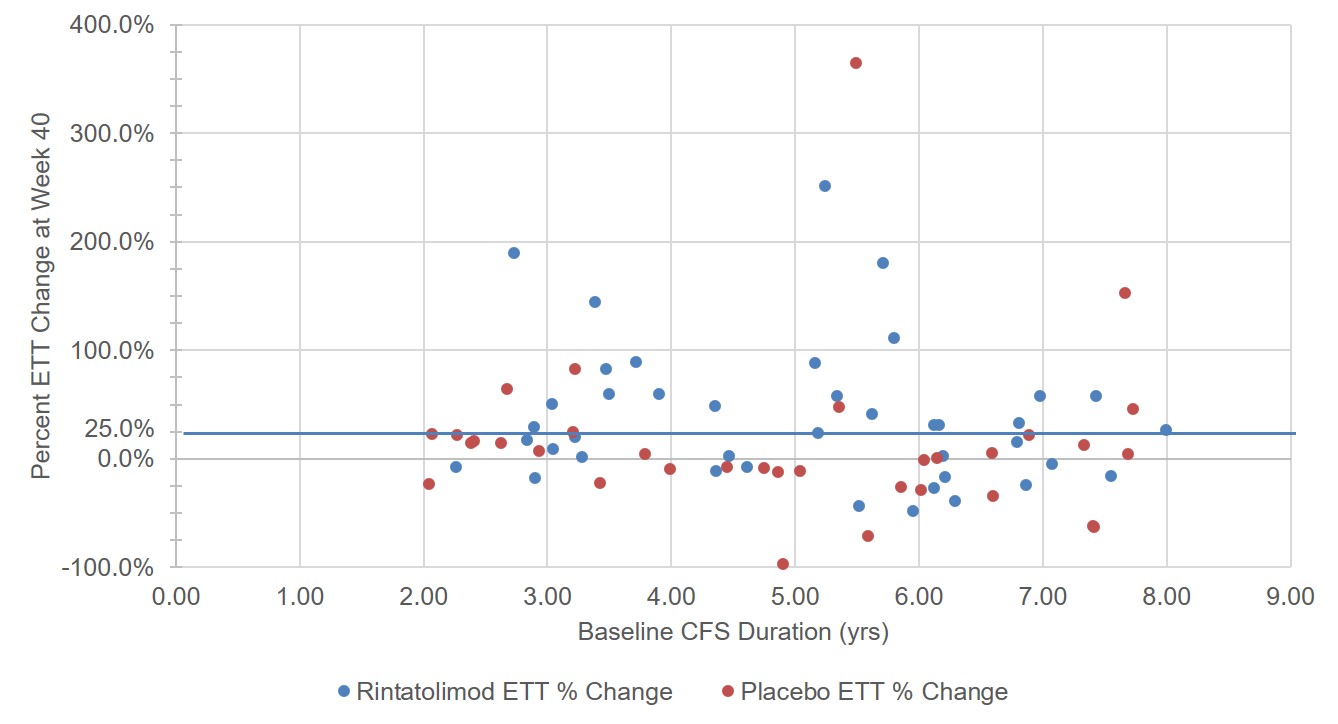


**Figure S1. Percent Change in ETT at Week 40 for the Target Subset based on Baseline CFS Duration**

Supplement: S1 Fig — (DOCX) [file pone.0240403.s001.docx]

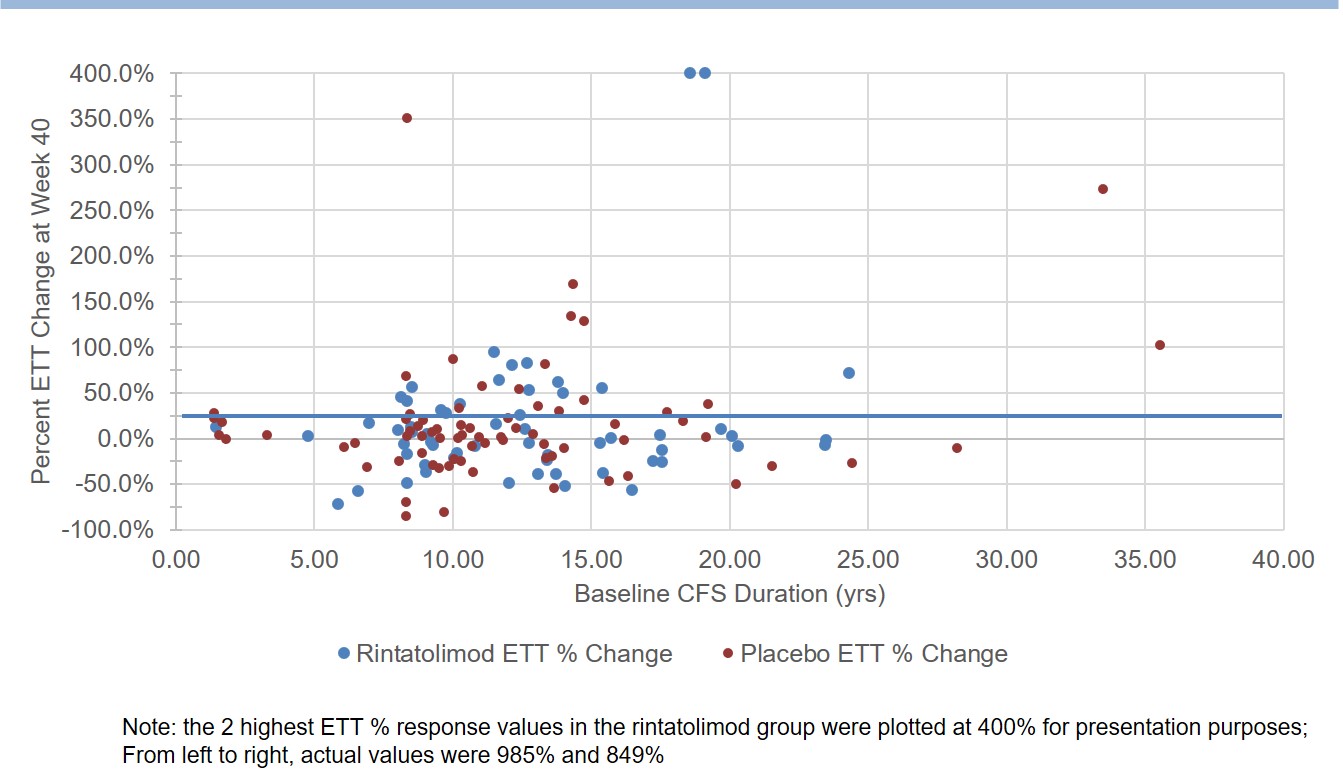


**Figure S2. Percent Change in ETT at Week 40 for the Non-Target subset based on Baseline CFS Duration**

Supplement: S2 Fig — (DOCX) [file pone.0240403.s002.docx]
