# Supplementary material for: Effect of disease duration in a randomized Phase III trial of rintatolimod, an immune modulator for Myalgic Encephalomyelitis/Chronic Fatigue Syndrome
Source: PLoS One. 2020 Oct 29;15(10):e0240403. doi: 10.1371/journal.pone.0240403 (PMC7595369; doi:10.1371/journal.pone.0240403)
Supplement: S1 Table — (DOCX) [file pone.0240403.s003.docx]

**Table S1.** **Comparative treadmill exercise testing protocols**

**A. Standard Bruce cardiovascular treadmill exercise testing protocol (13)**

| Stage | Minutes of Exercise (Elapsed) | Grade  % | Grade Increment % | Belt Speed  (MPH) | Increment  (MPH) |
| --- | --- | --- | --- | --- | --- |
| I | 0-3 | 10 | - | 1.7 | - |
| II | 3-6 | 12 | 2 | 2.5 | 0.8 |
| III | 6-9 | 14 | 2 | 3.4 | 0.9 |
| IV | 9-12 | 16 | 2 | 4.2 | 0.8 |

**B.** **ME/CFS treadmill exercise testing protocol (12)**

| Stage | Minutes of Exercise (Elapsed) | Grade  % | Grade Increment  % | Belt Speed  (MPH) | Increment  (MPH) |
| --- | --- | --- | --- | --- | --- |
| I | 0-2 | 0 | - | 2 | - |
| II | 2-4 | 3 | 3 | 2 | 0 |
| III | 4-6 | 6 | 3 | 2 | 0 |
| IV | 6-8 | 9 | 3 | 2 | 0 |
| V | 8-10 | 12 | 3 | 2 | 0 |
| VI | 10-12 | 15 | 3 | 2 | 0 |
| VII | 12-14 | 18 | 3 | 2 | 0 |
| VIII | 14-16* | 21 | 3 | 2 | 0 |
| IX | 16-18 | 21 | 0 | 3 | 1 |
| X | 18-20 | 21 | 0 | 4 | 1 |
| XI | 20-22 | 21 | 0 | 5 | 1 |
| XII | 22-24 | 21 | 0 | 5 | 0 |

*Maximal duration (16 minutes) for admission to AMP-516

**C.** **Debilitated/ elderly (Naughton) cardiovascular treadmill exercise testing protocol (14)**

| Stage | Minutes of Exercise (Elapsed) | Grade  % | Grade Increment  % | Belt Speed  (MPH) | Increment  (MPH) |
| --- | --- | --- | --- | --- | --- |
| I | 0-3 | 0 | - | 2 | - |
| II | 3-6 | 3.5 | 3.5 | 2 | 0 |
| III | 6-9 | 7.0 | 3.5 | 2 | 0 |
| IV | 9-12 | 10.5 | 3.5 | 2 | 0 |
| V | 12-15 | 14.0 | 3.5 | 2 | 0 |
