# Supplementary material for: Effect of disease duration in a randomized Phase III trial of rintatolimod, an immune modulator for Myalgic Encephalomyelitis/Chronic Fatigue Syndrome
Source: PLoS One. 2020 Oct 29;15(10):e0240403. doi: 10.1371/journal.pone.0240403 (PMC7595369; doi:10.1371/journal.pone.0240403)
Supplement: S2 Table — (DOCX) [file pone.0240403.s004.docx]

**Table S2. Clinical significance of Karnofsky Performance Scale* scores**

| **Score Description** | |
| --- | --- |
| 100 | Normal activity; no complaints; no evidence of disease. |
| 90 | Able to carry on normal activity; minor signs or symptoms of disease. |
| 80 | Normal activity with effort; some signs or symptoms of disease. |
| 70 | Cares for self, unable to carry on normal activity or do active work. |
| 60 | Requires occasional assistance but is able to care for most of needs. |
| 50 | Requires considerable assistance for daily care. |
| 40 | Disabled; unable to care for self, requires special care and assistance. |
| 30 | Severely disabled; bedridden although death is not imminent. |
| 20 | Very sick; hospitalization and/or nursing care is necessary; active support treatment is necessary. |
| 10 | Moribund; fatal processes progressing rapidly. |
| 0 | Dead. |

* KPS is a physician assessment of disability. Used as an assessment of group status, smaller shifts are expected than individual responses to disease intervention. A KPS of 40 to 60 was required for AMP-516 admission.
